# An Efficient PCA-GA-HKSVM-Based Disease Diagnostic Assistant

**DOI:** 10.1155/2021/4784057

**Published:** 2021-10-20

**Authors:** Brenda Jerop, Davies Rene Segera

**Affiliations:** Department of Electrical and Information Engineering, University of Nairobi, Kenya

## Abstract

Disease diagnosis faces challenges such as misdiagnosis, lack of diagnosis, and slow diagnosis. There are several machine learning techniques that have been applied to address these challenges, where a set of symptoms is applied to a classification model that predicts the presence or absence of a disease. To improve on the performance of these techniques, this paper presents a technique which involves feature selection using principal component analysis (PCA), a hybrid kernel-based support vector machine (HKSVM) classification model and hyperparameter optimization using genetic algorithm (GA). The HKSVM in this paper introduces a new way of combining three kernels: Radial basis function (RBF), linear, and polynomial. Combining local (RBF) and global (linear and polynomial) kernels has the effect of improved model performance. This is because the local kernels are better able to distinguish points closer to each other while the global kernels are more suited to distinguish points that are far away from each other. The PCA-GA-HKSVM is used on 7 different medical datasets, with two datasets being multiclass datasets and 5 datasets being binary. Performance evaluation metrics used were accuracy, precision, and recall. It was observed that the PCA-GA-HKSVM offered better performance than the single kernel support vector machines (SVMs).

## 1. Introduction

The fourth industrial evolution has come with big data, artificial intelligence (AI), and powerful machines with high processing power. This has resulted in the rise of the utilization of machine learning (ML) to solve complex problems. ML is a field of artificial intelligence (AI) that involves using algorithms to find patterns in data. One area that could benefit greatly from this advancement is the medical field. More specifically, ML has the ability to improve the field of disease diagnosis.

One challenge in disease diagnosis is misdiagnosis. This is a very common medical error that has been made by both inexperienced and expert doctors [1]. A major contributor to misdiagnosis is that symptoms vary per patient, and some patients present symptoms that are medically unexplained [2]. This makes it difficult to come up with a set of all the possible rules that define the pattern of a disease. Another challenge is lack of diagnosis. One factor that contributes to this is that many diseases have similar symptoms, and some symptoms are so mild that they are regarded as common signs of aging or normal discomforts. Hence, many people are hesitant to seek medical attention. Another factor is cost [3]. Many people cannot afford to obtain proper diagnosis. Thus, they may self-medicate without the guidance of a doctor. Slow diagnosis is another challenge. There are few specialists and sometimes, people have to wait a long time in order to obtain a diagnosis. This is dangerous because the disease could progress to later stages, making treatment more difficult [4].

Fortunately, more medical data is available now more than ever. There is a lot of data depicting a set of symptoms and their correct diagnosis collected through methods such as consultation, blood tests, and imaging tests. Applying this data to ML models could help to address the challenges mentioned above. It can result in faster diagnosis and less cases of misdiagnosis by providing decision support to doctors [5]. There are many studies that have applied ML techniques to the problem of disease diagnosis. These studies have shown that ML techniques can provide a reliable decision support system for disease diagnosis, especially in situations where specialists are few, or when there is difficulty in obtaining a definitive diagnosis [6].

Support vector machines have been shown to be a highly effective ML model in the field of disease diagnosis. For instance, Burbidge et al. [7] performed a comparative study of SVM with other machine learning techniques. The benchmark test was performed using data obtained from the UC Irvine machine learning repository to predict the inhibition of dihydrofolate reductase by pyrimidines. Their results showed that SVM outperformed a radial basis function network, a decision tree, and three artificial neural networks. Battineni et al. [8, 9] conducted a review of how machine learning is used in chronic disease diagnosis. The analysis involved a review of 453 papers published between 2015 and 2019. While they concluded that there was no single best approach for ML in disease diagnosis, they found that logistic regression and SVMs were the most commonly used and were highly applicable in disease diagnosis.

Jegan [10] presented a disease diagnosis system based on SVM. They found that the SVM achieved higher accuracy compared to other techniques. Safdar et al. [11] used RBF kernel in SVM for diabetes diagnosis. Using the Pima Indian diabetes dataset, 78% accuracy was achieved. Mohan [12] analyzed several studies presenting decision support systems that diagnosed heart disease. In the results, myocardial infarction was classified by artificial neural networks (ANN) with 97% accuracy and myocardial perfusion scintigraphy with 87.5% accuracy. A heart failure dataset was classified by a classification and regression tree (CART) with 87.6% accuracy. Heart valves were classified by neural network ensembles classified heart with 97.4% accuracy. SVM was able to classify arrhythmia screening resulting in 95.6% accuracy. Logistic regression managed to classify acute coronary syndrome with an accuracy of 72%. From this, it is seen that SVM achieved high accuracy. Trambaiolli et al. [13] employed SVMs and Naive Bayes classification algorithms in predicting liver disease. The dataset used was ILPD obtained from UCI, comprising 560 instances and 10 attributes. The SVM achieved an accuracy of 79.66% in 3210.00 ms while the correctness of Naive Bayes (NB) was 61.28% in a duration of 1670.00 ms. From this, it can be seen that SVM achieved higher accuracy. However, it took more time compared to NB. Alam et al. [14] used SVM in diagnosing Alzheimer's. They achieved an 87.0% accuracy and 91.7% sensitivity.

From these studies, it can be seen that SVMs are highly effective in classification of symptoms. The performance of SVMs heavily depends on the type of kernel used. Several researchers have shown that techniques that combine more than one kernel offer better performance and less occurrences of overfitting than single-kernel SVMs. Keerthika and Premalatha [15] presented an approach to differentiate between patients with Alzheimer's disease and healthy controls. PCA and a multikernel SVM was employed, yielding 84% stratification accuracy, and sensitivity and specificity above 85%. The multikernel SVM performed better than the linear kernel SVM. Shahbakhti et al. [16] proposed a cardiovascular disease diagnosis assistant based on efficient feature selection and classification using HKSVM. Their model resulted in an accuracy of 96.03% on the Cleveland dataset which is available at the UCI ML repository. Tuysuzoglu and Birant [17] presented Enhanced Bagging (eBagging) as a novel approach for ensemble learning. The technique was applied to SVM on several medical datasets. Their findings showed that eBagging produced better results than a single SVM.

It has also been shown that using feature selection techniques offers better performance. Wu and Zhou [18] presented a technique that used PCA for feature extraction and SVM for classification. The PCA was able to extract 3 optimized features from the dataset and a classification accuracy of 91.5%. Rubini and Perumal [19] used PCA in combination with SVM for cervical cancer diagnosis. The cervical cancer data contained 32 risk factors and 4 target variables: Hinselmann, Schiller, Cytology, and Biopsy. The targets were classified using SVM, SVM with recursive feature elimination, and SVM with PCA (PCA-SVM). PCA-SVM proved superior to the other two. Rubini and Perumal [20] suggested a chronic kidney disease diagnosis approach based on grey wolf optimization (GWO) for choosing the best features and HKSVM for classification. The outcome was 97.26% accuracy for the chronic kidney dataset on UCI ML repository. Tan et al. [21] proposed a technique that involved a multikernel support vector machine (MKSVM) together with fruit fly optimization algorithm (FFOA) for classification of diseases. The FFOA was used to find the best features for prediction. This approach attained a precision of 98.5% on chronic kidney dataset, 89.11565% on Hungarian chronic kidney disease dataset, 90.42904% on Cleveland chronic kidney disease dataset, and 86.17886% on Switzerland chronic kidney disease dataset from the UCI Machine Learning repository.

In order to improve the performance of the classification models, several hyperparameter optimization techniques have been shown to be successful. Santhanam and Padmavathi [22] proposed a disease diagnosis technique involving GA and SVM. The technique was applied on five datasets of different diseases from the UCI ML repository. The accuracies obtained were 84.07% for heart disease, 78.26% for diabetes, 76.20% for breast cancer, and 86.12% for hepatitis. Segera et al. [23] proposed a medical diagnosis approach based on GA and SVM. The approach was applied to the Pima Indians Diabetes from UCI repository. This technique achieved an accuracy of 98.79%, which is higher than 96.71% achieved from SVM with data preparation based on *K*-means clustering.

Few researchers have used all three techniques together (feature selection, hybrid kernels, and hyperparameter optimization). Ali et al. [24] presented a particle swarm optimized hybrid kernel-based multiclass support vector machine for microarray cancer data analysis. He proposed a novel linear-Gaussian-polynomial (LGP) hybrid kernel-based SVM for multiclass disease diagnosis. Dimensionality reduction was also applied to the datasets used. This PSO-PCA-LGP-MCSVM configuration achieved high accuracy compared to existing techniques. The technique was applied to microarray cancer datasets. Saseendran et al. [25] proposed a liver cancer diagnosis system based on linear discriminant analysis (LDA) for dimensionality reduction, SVM for classification, and GA for hyperparameter optimization. The method showed improved performance and lower training time. The method achieved 90.30% accuracy, 82.25% sensitivity, 96.07% specificity, and 0.804 Matthews Correlation Coefficient (MCC).

From the above studies, it can be seen that while ML has been successfully applied to the problem of disease diagnosis, there are few researchers that have applied feature selection, HKSVM, and hyperparameter optimization techniques to binary and multiclass diagnosis datasets all in one study. This paper is aimed at bridging that gap by using PCA for feature selection, using a HKSVM as a classification model, and using GA for hyperparameter optimization.

This paper contains an introduction of the problem and the solution as shown in this section.  Section 2 explores machine learning concepts while Section 3 contains a detailed explanation of the PCA-GA-HKSVM technique that has been proposed.  Section 4 explores the datasets that the technique was applied to.  Section 5 presents the results obtained and discusses them.  Section 6 gives a conclusion based on the results and presents recommendation for further work.

## 2. Machine Learning

Machine learning (ML) is a field of artificial intelligence (AI) that involves using algorithms to find patterns in data. This is in contrast with traditional programming (TP) where all the possible rules for a pattern have to be explicitly programmed and tested before the algorithm is used. For cases where there are many rules, the TP approach can be difficult to implement and maintain. Hence, ML can be more suitable than TP in certain aspects:

- Complex problems where the rules that define a pattern require a lot of fine tuning
- Problems in which the rules that define a pattern are too many hence making it difficult to maintain a TP approach
- Problems where the rules that define a pattern are constantly changing
- Problems where the rules are not known completely, and it is desired to get insights about the patterns

Disease diagnosis is one of the areas that is suited for ML. Many diseases have similar symptoms, and symptoms vary greatly between patients. A TP approach would be incredibly tedious since it would require a lot of fine tuning.

### 2.1. Exploratory Data Analysis and Transformation

This involves analyzing the dataset in order to get insights about it and transforming the data based on the insights. Data is analyzed for factors like:

- Leakage: this is when the training data includes information that will not be available during inference. This could lead to a false sense of confidence in the model performance
- Correctness: incorrect data could arise due to factors like human error during data collection
- Data types: model training involves matrix multiplication; hence, string data needs to be converted to numerical before being fed to the model
- Missing data: these need to be either removed or replaced before the data is fed into the model
- Class distribution: the data should contain sufficient instances from all classes

### 2.2. Data Splitting

When an ML algorithm is applied to a dataset, the result is usually an equation that represents the pattern in the dataset. This is known as a machine learning model. A machine learning dataset is usually split into a training set, validation set, and test set. Before splitting, the dataset is shuffled in order to ensure random class distribution, hence less chances of bias in the model.

### 2.3. Model Training

Model training involves fitting the algorithm on the training set so as to find the optimal parameter values for the model. If the model is too complex or the training contains noise, too few features or instances, the model may learn the peculiarities in the noise and will result in poor generalization on unseen data. This is known as *overfitting.*

There are several ways to avoid overfitting:

- Simplifying the model by constraining it. This is known as regularization
- Increasing the size of the train data
- Removing outliers and errors in the train data to reduce noise and peculiarities

On the other hand, the model may also underfit on the training data. This occurs when the model is so simple that it is not able to learn the patterns in the data.

To fix underfitting:

- Use a more complex model
- Reduce the regularization hyperparameter
- Add more predictive features to the algorithm
- Add more instances to the dataset

### 2.4. Model Validation

In order for a model to be useful, it needs to perform well on unseen data. A portion of unseen data is usually held out for the purpose of validation. This is known as a validation set. A validation set is helpful to ensure that the best learning algorithm and hyperparameter values have been chosen.

### 2.5. Crossvalidation

In many practical cases, there may not be enough data to allow holding out a portion for validation. If a portion of the data is held out for validation, there may not be enough for the model to train on; hence, the model will not have good learning ability. In such cases, a helpful technique would be one known as *crossvalidation*.

Crossvalidation involves splitting the dataset into *k* subsets of a similar size, where *k* is the number of subsets. Each subset is referred to as a fold. A common example is where the dataset is split into five folds: {*F1*, *F2*, *F3*, *F4*, *F5*}. This is known as fivefold crossvalidation. Five models are then trained. The first model *f1* is trained on folds *F2*, *F3*, *F4*, and *F5*, and fold *F1* is used as a validation set. Similarly, the second model *f2* is trained on folds *F1*, *F3*, *F4*, and *F5*, and fold *F2* is used as a validation set. The same pattern applies to models *f3*, *f4*, and *f5*. The evaluation metric is calculated for each model, and an average is taken to obtain the final value.

Once the optimal hyperparameter values have been found, the entire training set can be used for training.

### 2.6. Testing

Testing is the last step before the model can be used for inference. Testing ensures that the model performs as expected. It involves applying the model to an unseen portion of the dataset not used for training or validation. The test set should be representative of the real-world data that the model will be used on.

## 3. PCA-GA-HKSVM

### 3.1. Principal Component Analysis (PCA)

Processing very large datasets is computationally intensive. There could also be correlated features that only add noise to the model and slow down the training [26]. Principal component analysis (PCA) is a technique introduced by Pearson [11] as a way to reduce the dimensionality of a dataset while preserving as much information as possible. It can be used to identify the minimum number of uncorrelated features. This results in faster training time, better model performance, lower computing costs during training, and easier data visualization using plots.

PCA works by finding the closest hyperplane to the data, then projecting the data onto it. The hyperplane should preserve maximum variance, because this means that it loses less information. After finding the axis that preserves maximum variance, it then finds a second axis that preserves the largest of the remaining variance. This second axis is orthogonal to the first. A third axis is then identified orthogonal to the previous two and so on until the number of axes is equal to the number of dimensions in the dataset. The *k*^th^ axis is referred to as the *k*^th^*principal component*. Singular value decomposition (SVD) is a matrix factorization method used to find the principle components. SVD decomposes the train set matrix **x** into a product of three matrices.

(1) $$\begin{array}{c} V=\left[\begin{array}{c} :\\ v1\\ : \end{array}\begin{array}{c} :\\ v2\\ : \end{array}\begin{array}{c} :\\ v3\\ : \end{array}\right], \end{array}$$

where *V* has the unit vectors of the principal components.

In order for the dimensionality of the data to be reduced to *k* dimensions, it should to be projected to a hyperplane that is defined by the first *k* components, resulting in a reduced training matrix **X**_reduced_. This is done by multiplying matrix **X** by matrix **V**_*d*_, which contains the first *d* columns of **V**.

(2) $$\begin{array}{c} X_{\text{reduced}}={XV}_{d}. \end{array}$$

In this paper, MATLAB's *pca* function [27] was used to perform PCA on the dataset.

### 3.2. Hybrid Kernel-Based Support Vector Machine

The concept of a support vector machine (SVM) was introduced by Vladimir Vapnik [28]. SVM is a machine learning algorithm that can be used to build a model that performs various types of predictions such as classification, regression, and outlier detection. It is mostly used on small- and medium-sized datasets. SVMs have the ability to capture large feature spaces because of their generalization principle based on structural risk minimization (SRM) [29].

For a case where data is two-dimensional, the SVM hyperplane is a line that separates the positive examples from the negative ones. The boundary that separates the examples is called the decision boundary. A linear SVM finds the equation of the decision boundary using the decision function *y*′ = **w**^⊺^**x** + *b*. If the result is greater than one, then the prediction is a positive class; otherwise, it is a negative class.

(3) $$\begin{array}{c} y^{\prime }=\left\{\begin{array}{c} 0,\;\text{if }w^{\text{T}}\text{x}+\text{b}\leq 0\\ 1,\;\text{if }w^{\text{T}}\text{x}+\text{b}\geq 0 \end{array}\right\}, \end{array}$$

where

- **w** = weight vector
- **x** = input feature vector
- *b* = bias

The points where the decision function is zero result in the formation of the decision boundary. The optimal hyperplane is one that separates the positive and negative examples with the largest margin. Training involves looking for the values of *b* and **w** that give this optimal hyperplane. This is so that the model can generalize well to future unseen examples.

In order to obtain the largest margin, the Euclidean norm of **w**, ‖**w**‖ has to be minimized. Hence, the optimization problem is

(4) $$\begin{array}{c} \mathrm{Min}\left\|w\right\|\text{ while }y_{i}\left({wx}_{i}–b\right)\geq 1,\;\text{ for }i=1\cdots N, \end{array}$$

where

- $\left\|w\right\|=\sqrt{\sum_{j=1}^{D}{\left(w^{\left(j\right)}\right)}^{2}}$
- *D* = number of dimensions

At the margin lines, the decision function is equal to either 1 or -1 The lines are equidistant and parallel to the decision boundary.

In most practical datasets, there is not a straight line that can separate the positive from the negative examples. In this case, the kernel trick is employed. This is by mapping the data to higher dimensions where the examples may be separable. For example, if there is a two-dimensional dataset that is not linearly separable, it can be transformed into three-dimensional linearly separable data by mapping *ϕ* : **x**⟶*ϕ*(**x**).

(5) $$\begin{array}{c} \phi \left(x\right)=\phi \left(\left(\begin{array}{c} x_{1}\\ x_{2} \end{array}\right)\right)=\left(\begin{array}{c} x_{1}^{2}\\ \sqrt{2}x_{1}x_{2}\\ x_{2}^{2} \end{array}\right), \end{array}$$

where **x** is a dataset of *n* dimensions.

However, the most appropriate mapping is not usually known beforehand. A solution would be to try all possible mapping functions then fitting a linear SVM but this is not computationally efficient.

An alternative and more efficient method is to use kernel functions, which allow working in higher dimensions without explicitly transforming the data. For instance, to transform a two-dimensional vector to three dimensions, it can be seen below that the square of the dot product of the initial two-dimensional vector can be found from the dot product of the transformed three-dimensional vector:

(6) $$\begin{array}{c} \phi {\left(a\right)}^{T}\phi \left(b\right)={\left(\begin{array}{c} a_{1^{2}}\\ \sqrt{2}a_{1}b_{2}\\ a_{2^{2}} \end{array}\right)}^{T}\left(\begin{array}{c} a_{1^{2}}\\ \sqrt{2}b_{1}b_{2}\\ b_{2^{2}} \end{array}\right)={\left(a^{T}b\right)}^{2}. \end{array}$$

From above, it can be seen that it is sufficient to replace the dot product of the transformed vectors by the square of the original vectors instead of explicitly transforming the data. Hence, a kernel is a function that can obtain the dot product using only the original vectors.

A single kernel may not be able to provide a full representation of the dataset. Even if that kernel exists, there is no standard method that can be applied to choose a kernel. Hence, a combination of multiple kernels may be employed to provide better learning ability, leading to improved performance [30].

There are different types of kernel functions. The most widely used are

(7) $$\begin{array}{c} \text{Linear}:K_{\text{linear}}=u^{T}v,\\ \text{Polynomial}:K_{\text{polynomial}}={\left(\gamma u^{T}v+r\right)}^{d},\\ \text{RBF}:K_{\text{rbf}}=\exp\left(-\gamma {\left\|u-v\right\|}^{2}\right),\\ \text{Sigmoid}:K_{\text{sigmoid}}=\tanh\left(\gamma u^{T}v+r\right). \end{array}$$

The hybrid kernel used in this project is a combination of the RBF, linear, and polynomial kernels.

Let:

*K*_1_ be a combination of RBF and linear kernel:

(8) $$\begin{array}{c} K_{1}={\gamma K}_{\text{linear}}-\left(1–\gamma \right)K_{\text{rbf}}. \end{array}$$

*K*_2_ be a combination of RBF and linear kernel:

(9) $$\begin{array}{c} K_{2}={\gamma K}_{\text{polynomial}}-\left(1–\gamma \right)K_{\text{rbf}}. \end{array}$$

*K*_3_ be a combination of linear and polynomial kernel:

(10) $$\begin{array}{c} K_{3}={\gamma K}_{\text{linear}}-\left(1–\gamma \right)K_{\text{polynomial}}. \end{array}$$

Then, the final hybrid kernel used in this project is:

(11) $$\begin{array}{c} K_{h}=\alpha_{1}K_{1}+\alpha_{2}K_{2}+\alpha_{3}K_{3}. \end{array}$$

With the following constraints:

(12) $$\begin{array}{c} \alpha_{1}+\alpha_{2}+\alpha_{3}=1,\\ \gamma <=1, \end{array}$$

where

- *α*_1_ = Coefficient for the linear-rbf hybrid kernel
- *α*_2_ = Coefficient for the polynomial-rbf hybrid kernel
- *α*_3_ = Coefficient for the linear-polynomial hybrid kernel
- *γ* = Coefficient for the single kernels (RBF, linear, polynomial)
- *K*_rbf_ = RBF kernel
- *K*_polynomial_ = Polynomial kernel
- *K*_linear_ = Linear kernel

The optimal values for *α*_1_, *α*_2_, *α*_3_, and *γ* are determined through hyperparameter optimization. The LIBSVM [31] library was used to train the hybrid model with the precomputed kernel option.

The RBF kernel is a local kernel. This means it was able to distinguish between points close to each other, resulting in a better learning ability of the model. The polynomial kernel and the linear kernel are global kernels. Hence, they were able to better distinguish between points far away from each other. This resulted in the model being able to generalize well on new data. Hence, a combination of local and global kernels prevents both underfitting and overfitting.

### 3.3. Hyperparameter Optimization

One factor that greatly affects how the model learns from the training data is the hyperparameters. Hyperparameter tuning involves training the model on different values of the hyperparameters, then choosing the ones that give the optimal performance.

There are several methods of tuning hyperparameters such as the grid-search technique. Genetic algorithm (GA) has been shown to perform better than faster [32].

GA falls under a computation technique known as evolutionary computation. It was developed at the University of Michigan by John Holland and his colleagues and students [17]. A population of possible solutions undergo mutation just like in natural genetics, resulting in children. The process is repeated as each individual is given a fitness value. Those with higher fitness values are used to produce results with even higher fitness values until a stopping criterion is reached. Starting with an initial population, new off-springs are generated by applying crossover or mutation operators to them. The new off-springs then replace the initial population, and the process is repeated.

MATLAB's *ga* function [33] was used as the genetic algorithm. Fivefold crossvalidation was used as a fitness function with the crossvalidation accuracy as the fitness value. Since the genetic algorithm tries to minimize the fitness value and the highest crossvalidation accuracy was of interest, the fitness value was a product of the crossvalidation accuracy and -1.

### 3.4. PCA-GA-HKSVM Process

**Figure 1:**
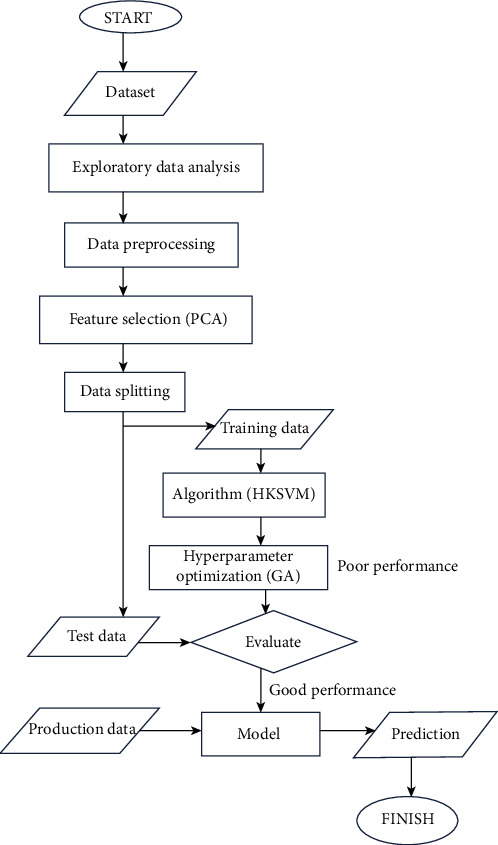
Flowchart of PCA-GA-HKSVM process.

The entire PCA-GA-HKSVM process was done in MATLAB R2018a on a Linux PC with 4 GB RAM. The flowchart is shown in Figure 1, and the process is outlined as follows:

(1) Load a dataset into MATLAB
(2) Explore the dataset to get insights about:
  - The data types of the features, whether numerical or categorical
  - The range of feature values
  - Presence of outliers, missing data, and bad quality data such as values out of range
  - Presence of correlated features
  - Class distribution
(3) Transform the data according to insights obtained in step (2). This involves:
  (vi) Encoding categorical features
  (vii) Imputing missing data
  (viii) Scaling the data
  (ix) Removing bad quality data points such as improbable values of age and temperature
  (x) Shuffling the dataset to ensure random class distribution
(4) Apply MATLAB's *pca* function to the dataset to obtain:
  (xi) The principal component coefficient matrix
  (xii) A matrix containing the principal component scores
  (xiii) A vector with the principal component variances
  (xiv) A vector with the percentage of the total variances explained by each principal component

The reduced matrix contains the principal components that account for at least 95% of the variance.

(5) Transform the data into LIBSVM format
(6) Split the data into train and test sets, with 80% for training and 20% for testing
(7) Compute the kernel matrix using the new HKSVM presented
(8) Use the negative 5-fold crossvalidation accuracy of the HKSVM as a fitness value in MATLAB's *ga* function to obtain the optimal coefficient values of the HKSVM
(9) Apply the entire training set to train the HKSVM with the optimal hyperparameter values returned in step (8) to obtain a model
(10) Apply the test data to the model and obtain the accuracy, precision, and recall of the model

## 4. Performance Evaluation

Several datasets were used to train and test the PCA-GA-HKSVM. The data was sourced from the UCI Machine Learning Repository and Kaggle.

The respiratory disease dataset [34] is a multiclass dataset containing 20 features and 44,454 instances which can help in distinguishing between cold, flu, COVID-19, and allergies. Its labeling was based on symptoms listed in the Mayo Clinic website. The class representation is heavily imbalanced, with one class containing 25,000 instances and another containing less than 2000 instances. The dataset was reduced to an average of 1000 instances per class in order to reduce bias.

The Chronic Kidney Disease dataset [35] is a binary dataset containing 400 instance and 24 features. The label class indicates the presence or absence of chronic kidney disease.

The Heart Disease dataset [36] is a subset of the UCI machine learning repository heart disease dataset that dates from 1988 and is from four databases: Cleveland, Hungary, Switzerland, and Long Beach V. It contains 303 instances, 13 attributes, and a target class to depict the presence or absence of heart disease.

The Breast Cancer Wisconsin dataset [37] contains features that are computed from a digitized image of a fine needle aspirate of a breast mass. There are 30 features and 569 instances. The target class depicts whether the mass is malignant or benign. Hence, the dataset can be used as guidance on whether there is a likelihood of a mass being cancerous.

The Lymphography dataset [37] contains observations provided by the Oncology Institute. It contains 148 instances with 18 attributes. The target contains four classes: normal, metastases, malignant lymph, and fibrosis.

The Acute Inflammation dataset [38] contains 6 attributes and 120 datasets. It was obtained from the UCI Machine Learning Repository. The target class of the dataset shows whether the features are indicative of either acute nephritis of renal pelvis origin or acute bladder inflammation.

### 4.1. Performance Evaluation Metrics

The performance of the model was evaluated based on several metrics. These are the following.

#### 4.1.1. Accuracy

The crossvalidation accuracy for each model was investigated. However, accuracy is usually not the most efficient performance evaluation metric in cases where one class was more frequent than others or where the cost of misclassification of one class was greater than another. Hence, more classification metrics were needed.

#### 4.1.2. Confusion Matrix

To get a better sense of the actual performance even in skewed datasets, a confusion matrix was obtained for each dataset. Number of instances where one class was classified as another was obtained and used to generate the matrix. Each row represents an actual class, while each column represents a predicted class. Negative classes that were correctly classified are called true negatives. On the other hand, negative classes that were wrongly classified are called false negatives. Positive classes that were correctly classified are called true positives. On the other hand, positive classes that were wrongly classified are called false positives. A perfect classifier only has true positives and true negatives; hence, its confusion matrix only contains nonzero values in its top-left to bottom-right diagonal.

#### 4.1.3. Precision

From the confusion matrix, the precision was obtained. This refers to the ratio of true positives to all the positive predictions made.

(13) $$\begin{array}{c} \text{precision}=\frac{\text{TP}}{\text{TP}+\text{FP}}, \end{array}$$

where

- TP = true positives
- FP = false positives

#### 4.1.4. Recall

Another metric that was obtained from the confusion matrix is the recall. This refers to the true-positive rate. It is the ratio of true positives in the predictions to actual positives in the dataset.

(14) $$\begin{array}{c} \text{recall}=\frac{\text{TP}}{\text{TP}+\text{FN}}, \end{array}$$

where

- TP = true positives
- FN = false negatives

## 5. Results and Discussion

### 5.1. Hyperparameter Optimization

Crossvalidation training was done on the models with genetic algorithm to find the optimal values for *α*_1_, *α*_2_, *α*_3_, and *γ* as described in Section 3. The obtained results are shown in Table 1.

From Table 1, it can be seen that in most datasets, the coefficient *rbf-linear*(*α*_1_) was the highest. In the Breast Cancer and Chronic Kidney Disease datasets, the effect of the *linear-rbf* kernel alone resulted in the highest crossvalidation accuracy. It can also be seen that in most datasets, the optimal value of the coefficient *γ* was closer to 1 than to zero.

### 5.2. Precision

From the confusion matrix, the following precision values were calculated for each class in each dataset.

From the precision values in Table 2, it can be seen that the PCA-GA-HKSVM had perfect precision in classes of some datasets. In all datasets, the precision was above 0.75.

### 5.3. Recall

From the confusion matrix, the following recall values were calculated for each class in each dataset.

From the recall values in Table 3, it can be seen that the PCA-GA-HKSVM had perfect recall in classes of some datasets. In all datasets, the recall was above 0.7.

### 5.4. Comparison with Single Kernel Models

The table below presents the accuracy of the PCA-GA-HKSVM in comparison with the single kernel models.

From the accuracy values in Table 4, it can be seen that the PCA-GA-HKSVM achieved more than 80% accuracy in all the datasets. It also out-performed the single-kernel SVMs. The testing accuracy values were similar to the crossvalidation accuracy values; hence, the PCA-GA-HKSVM did not overfit on the data.

The *friedman()* function in MATLAB was applied in order to conduct Friedman's test on the accuracies obtained from 10 runs of 5-fold crossvalidation for each model and dataset.  Table 5 shows that the *p* values obtained for each dataset were all above 0.05, which is the maximum desired value in order for the null hypothesis to be rejected.

From Table 6, it can be seen that the PCA-GA-HKSVM has the longest training time. This is because of the additional PCA-GA steps in the process.

## 6. Conclusion

The main objectives of this project were to develop a new and effective hybrid kernel-based SVM for disease diagnosis and to use the model in conjunction with feature selection and hyperparameter optimization. The following were achieved:

- Feature selection of disease data using PCA: it was observed that selection of the most important features helped to reduce the training time of the model without reducing the accuracy
- The creation of a new hybrid kernel based model using a combination of local and global kernels: it was observed that the resulting kernel offered satisfactory performance in disease diagnosis using different datasets
- Hyperparameter optimization of the created HKSVM using GA: while this slowed down the training process, it was quite efficient in finding hyperparameter values that improved the accuracy of the model
- Performance evaluation for the new PCA-GA-HKSVM technique: the PCA-GA-HKSVM technique generally had higher accuracy than the single-kernel SVMs

Several drawbacks were also observed. This includes slow training time and high *p* value from Friedman's test. Further work could involve more advanced vectorization techniques to improve on running time and training on larger datasets.

## Figures and Tables

**Table 1:** Results of hyperparameter optimization.

Dataset	Crossvalidation accuracy	*α*_1_	*α*_2_	*α*_3_	*γ*
Respiratory diseases	96.31%	0.2208	0.3938	0.3855	0.2275
Nephritis	100%	0.3950	0.4253	0.1797	0.1615
Acute bladder inflammation	98.97%	0.4117	0.4579	0.1304	0.7639
Breast cancer	94.94%	1	0	0	1
Chronic kidney disease	95.94%	1	0	0	1
Lymphography	86.44%	0.8635	0.1365	1.3878e-17	0.4877
Heart disease	80.97%	0.949	0.0075	0.0075	1.00

**Table 2:** Precision values for the different datasets.

Dataset	Class 1	Class 2	Class 3	Class 4
Respiratory	1.0000	0.8932	1.0000	0.9725
Lymphography	0.8	0.8571	0.8221	0.8081
Nephritis	1	1	—	—
Acute bladder inflammation	1	1	—	—
Breast cancer	0.9559	0.8913	—	—
Chronic kidney disease	1	0.9074	—	—
Heart disease	0.8427	0.7759	—	—

**Table 3:** Recall values for the different datasets.

Dataset	Class 1	Class 2	Class 3	Class 4
Respiratory	0.9204	1.0000	0.9697	0.9636
Nephritis	1	1	—	—
Acute bladder inflammation	1	1	—	—
Breast cancer	0.9286	0.9318	—	—
Chronic kidney disease	0.8387	1	—	—
Lymphography	0.8571	0.8	0.8621	0.7936
Heart disease	0.7426	0.8654	—	—

**Table 4:** Accuracy of PCA-GA-HKSVM vs. single kernel SVMs.

Dataset	PCA-GA-HKSVM	RBF	Linear	Polynomial
Respiratory diseases	96.3547%	93.81%	93.05%	75.13%
Nephritis	100%	98%	100%	98%
Bladder inflammation	100%	98%	100%	98%
Breast cancer	92.98%	65.54%	87.61%	89.11%
Chronic kidney disease	93.7500%	92.21%	90.63%	92.74%
Lymphography	82.8576%	69.41%	79.76%	79.76%
Heart disease	80.49%	80.49%	77.64%	69.414%

**Table 5:** *p* value from Friedman's test.

Dataset	*p* value
Respiratory diseases	0.0952
Nephritis	0.2610
Bladder inflammation	0.1251
Breast cancer	0.0537
Chronic kidney disease	0.0825
Lymphography	0.0943
Heart disease	0.0621

**Table 6:** Running time in seconds.

Dataset	PCA-GA-HKSVM	RBF	Linear	Polynomial
Respiratory diseases	61.456	2.182	31.27	2.378
Nephritis	13.616	0.545	0.675	0.598
Bladder inflammation	3.480	0.533	0.532	0.512
Breast cancer	62.737	0.800	2.233	6.557
Chronic kidney disease	58.228	0.708	2.671	3.903
Lymphography	5.563	0.358	0.999	0.472
Heart disease	47.759	0.798	3.050	0.648

## Data Availability

All the data used in the preparation of this paper are available from the authors upon request.
